# An Experimental and FE Modeling Investigation of the Pull-Out Behavior of Anchoring Solutions in Concrete: A Comparative Study

**DOI:** 10.3390/ma18194596

**Published:** 2025-10-03

**Authors:** Alexandru-Nicolae Bizu, Dorina Nicolina Isopescu, Gabriela Draghici, Igor Blanari

**Affiliations:** 1 Faculty of Civil Engineering and Building Services, Gheorghe Asachi Technical University of Iasi, 700050 Iasi, Romania; alexandru-nicolae.bizu@student.tuiasi.ro; 2 Faculty of Civil Engineering, Ovidius University of Constanta, 900527 Constanta, Romania; gddraghici@gmail.com; 3 Faculty of Mechanics, Gheorghe Asachi Technical University of Iasi, 700050 Iasi, Romania; igor.blanari@academic.tuiasi.ro

**Keywords:** adhesive anchor, pull-out, finite element method (FEM), concrete, strengthening

## Abstract

This article presents an original experimental and numerical approach to examining the pull-out behavior of fastening systems made of steel bars simultaneously embedded in both ends of concrete samples. This double-embedded configuration simulates a connection between the existing concrete structure and a new external exoskeleton, promoting seismic strengthening. Pull-out tests were performed across six specimen configurations combining different concrete strength classes in order to compare the adhesive solution against traditional monolithic cast-in rebar embedments. The adhesive-anchored bars achieved a peak pull-out force of ~28.6 kN, which is about 18% higher than with mixed anchorage (one end adhesive, one end cast-in). All specimens failed in concrete cracking and pull-out cone formation, with no steel bar yielding, indicating that failure was governed by concrete strength. Finite element simulations in ANSYS Explicit Dynamics were validated against these experiments, confirming the observed behavior and enabling the extension of our analysis to broader concrete strength ranges. Overall, the results demonstrate that double-ended adhesive anchorage significantly increases the connection’s load-bearing capacity and ductility compared to mixed configurations.

## 1. Introduction

In the context of increasing seismic safety requirements for existing building stock, structural strengthening interventions are becoming increasingly important, particularly for reinforced concrete buildings constructed prior to the introduction of modern design codes. One of the effective solutions applied in recent years is strengthening through reinforced concrete exoskeletons, which allow external intervention without interrupting the building’s functionality. A critical aspect in implementing such solutions is the achievement of efficient structural connections between the exoskeleton and existing building, especially through reinforcing bars that are subsequently introduced. Due to their ability to transfer loads without expansion effects, even under challenging space or exposure conditions, adhesive anchors have emerged as a high-performance post-installed fastening technology [1,2,3]. Their behavior is governed by adhesive–steel–concrete interactions and influenced by numerous factors, such as concrete class, hole geometry, embedment depth, and installation conditions [3,4,5]. Beyond these parameters, the properties of both the concrete and reinforcing bars can significantly affect anchorage performance. For instance, incorporating steel fibers into the concrete mix has been shown to increase the pull-out capacity of anchors compared to plain concrete [6,7]. Likewise, using alternative concrete compositions (e.g., geopolymer concrete with partially or fully recycled aggregates) can alter bond stress development between steel and concrete [8,9]. On the reinforcement side, authors studying Fiber-Reinforced Polymer (FRP) bars have found that bar surface morphology is a critical factor governing bond strength [10,11]. Furthermore, novel rebar designs—such as helix-pultruded CFRP bars—have demonstrated substantially higher pull-out strengths (over 2.5× increase) thanks to improved mechanical interlocking with the concrete [12,13]. Although classical studies have extensively investigated anchor performance in standard configurations, there are limited data available regarding their behavior in situations where the bar is embedded at both ends, as frequently encountered in exoskeleton strengthening [2,14].

Adhesive anchors represent a post-installed fastening system in which a resin-based adhesive compound is used to insert and secure a metal rod (such as a threaded or reinforcing bar) into a solid base material, such as concrete or masonry. In contrast to mechanical anchors (e.g., expansion or screw types), which transfer forces through mechanical interlock, adhesive anchors operate through adhesion: the injected adhesive penetrates the irregularities and porosity of the substrate, ensuring a strong bond across the entire contact surface between the adhesive and rod after curing. Free of expansion effects, this mechanism reduces the risk of base material cracking and allows anchors to be placed near edges or in close proximity to one another (an option that is restricted in the case of mechanical anchors). In the literature, these systems are also referred to as bonded or adhesive anchors and are classified within the category of post-installed anchors, as opposed to pre-installed ones, which are simultaneously cast with the concrete. A typical adhesive anchoring system consists of a two-component adhesive (resin and hardener) and metallic fastening element. The main types of adhesives include polyester, vinylester (epoxy acrylate), and pure epoxy resins, each with specific applications: polyesters are frequently used for light anchoring and masonry; vinylesters provide higher strength and rapid curing; and pure epoxies exhibit excellent performance even under high-temperature conditions or when exposed to aggressive chemicals. From a packaging perspective, adhesives can be supplied in capsule form (glass or plastic) or as injectable cartridges. With the former, mixing occurs in situ through rod rotation, while injectable systems use a static mixer that ensures automatic blending at the time of application. Once the adhesive is introduced into the drilled hole, the rod is inserted, and compound curing leads to solid fixation. If properly designed, the anchor may exhibit ductile behavior, failing through metallic rod fracture rather than adhesion loss. Beginning in the 1970s, adhesive anchors were widely introduced, and have since become preferred solutions across multiple construction applications, both in new projects and in strengthening or extension works. They are used for post-installed bars in existing reinforced concrete elements (overlays, structural extensions), for equipment installation or façade element fixings, and in seismic retrofitting interventions where the integration of new elements requires reliable connections with the existing structure. Their versatility makes them suitable for anchoring in cracked or uncracked concrete, natural stone, masonry, or even in challenging conditions (humidity, low temperatures, marine exposure), by employing thixotropic formulations or certified products for special applications. They are also commonly used for mounting brackets, railings, or suspended equipment, where geometry or loading prohibits the use of mechanical anchors. The embedment depth can be tailored to structural requirements, circumventing the limitations of prefabricated components. A relevant example is Sika AnchorFix-2+, a two-component epoxy acrylate system intended for medium- to heavy-duty applications. According to its technical documentation, it is suitable for anchoring in cracked and uncracked concrete, natural stone, or steel, and benefits from a European Technical Assessment (ETA) issued under ETAG 001—Parts 1 (Anchors in General) and 5 (Bonded Anchors); these documents have since been withdrawn and replaced by EAD 330232-00-0601 and EAD 330499-01-0601. This type of product provides a documented performance (characteristic load-bearing capacities, reduction factors for various conditions) and is accompanied by detailed installation instructions, ensuring durability over the full service life, provided the manufacturer’s recommendations are strictly followed.

The importance of adhesive fastening solutions in structural engineering has led to extensive research on their performance under various conditions. Experimental studies have investigated typical failure modes (concrete cone pull-out, steel bar fracture, or combined failure), as well as the influence of geometric and material parameters on bond strength [1,15]. Other researchers have addressed anchor behavior under seismic loading, shear forces, and sustained loads. A notable synthesis was conducted by the authors of [2], who developed design methodologies and calibrated numerical models for adhesive anchors. Recent studies also examine performance in aged or low-strength concrete, behavior at extreme temperatures, and even non-destructive inspection methods for installed anchors [3]. These efforts laid the groundwork for standardized design provisions. Initially, guidelines like ETAG 001—Annex C [4] and TR 029 [3] provided design rules for bonded anchors; today, this knowledge is consolidated in EN 1992-4:2019 [5]. In parallel, American provisions (e.g., ACI requirements [16]) impose strict installation procedures, quality control, and operator training (according to standards such as [14]) measures prompted by failures like the Boston ‘Big Dig’ tunnel anchor collapse (2006), which was caused by adhesive bond failure under sustained load.

Adhesive anchors thus represent modern, adaptable, and efficient structural fastening solutions, provided that technical and installation requirements are rigorously respected. Their proper use depends on careful system type selection, adequate design, and appropriate application in accordance with current national and international standards.

In the literature, the pull-out (bond–slip) behavior of reinforcing bars has been extensively analyzed, with emphasis on the force–slip relationship, loading type (monotonic vs. cyclic), and bar surface conditions. A seminal study by [9,17] investigated bond development mechanisms under various loading regimes, laying the foundation for conceptual models of local bond failure. Subsequently, the authors of [18,19] proposed a ‘two-end pull-out test’ methodology in which a rebar is embedded in two concrete blocks simultaneously, with deformations monitored independently at each end using LVDT transducers. Although such two-sided anchorage experiments are rare, they provide valuable insights into stress distribution and pull-out mechanics in complex connections.

The present paper proposes an experimental and numerical investigation of the pull-out behavior of Ø14 mm reinforcing bars, anchored either by adhesive bonding or monolithically at both ends, in cubic concrete specimens of various strength classes. Our study reproduces a technically relevant situation in seismic strengthening, where a bar provides the connection between the existing and newly added structures. By testing six distinct configurations, the influence of concrete strength class and anchoring type on pull-out resistance and failure mode is assessed. The overall objective is to highlight the use of adhesive anchors as a reliable solution for seismic strengthening through a structural exoskeleton, as well as to propose an alternative testing methodology applicable to the evaluation of structural connections, as a complement to current international standard provisions [3,5]. Based on the available information, this double-embedded reinforcement experimental approach has not been documented in the literature and no direct precedents were found in international publications, underlining the originality of the testing model proposed.

## 2. Materials and Methods

### 2.1. Experimental Joint Assembly Configuration and Instrumentation

In the present study, the pull-out behavior of steel reinforcing bars is analyzed under special double embedment conditions, in which each end of the bar is anchored in a separate concrete element. The purpose of the experimental investigation is to highlight the differences in pull-out load-bearing capacity between the following two types of anchorage for Ø14 mm bars in concrete:

- Monolithic embedment: integrating the bar during concrete casting, achieving direct incorporation into the fresh concrete mass;
- Adhesive anchorage: subsequently inserting the bar into a core drilled in hardened concrete, followed by epoxy adhesive injection for fixation.

Since no standard test protocol exists for a bar anchored at both ends, the experimental procedure was developed by drawing inspiration from the classical pull-out test method commonly used to assess rebar–concrete bond strength [15]. In this study, this traditional configuration was modified such that the steel bar is simultaneously embedded and anchored at both ends in two separate concrete elements.

This experimental situation reflects a case applicable to engineering practice, namely the creation of a structural connection between an existing reinforced concrete element and a newly added, strengthening structure, such as a reinforced concrete exoskeleton. Such connections can typically be achieved either through monolithic reinforcement continuity between the existing and new structures, or by using post-installed adhesive anchors. Therefore, the main objective of the tests is to compare the performance of the two anchorage types, in terms of pull-out resistance and the corresponding structural behavior, under varying concrete strength classes.

According to this method, the bar is embedded at one end in concrete (in the form of a cube or cylinder), while an axial extraction force is applied at the opposite end. The bar is clamped in the testing machine grip, while the concrete element is supported, thereby allowing us to measure the force required to break the steel–concrete bond.

In this study, the standard configuration was modified so that the bar was simultaneously anchored at both ends.

**Figure 1:**
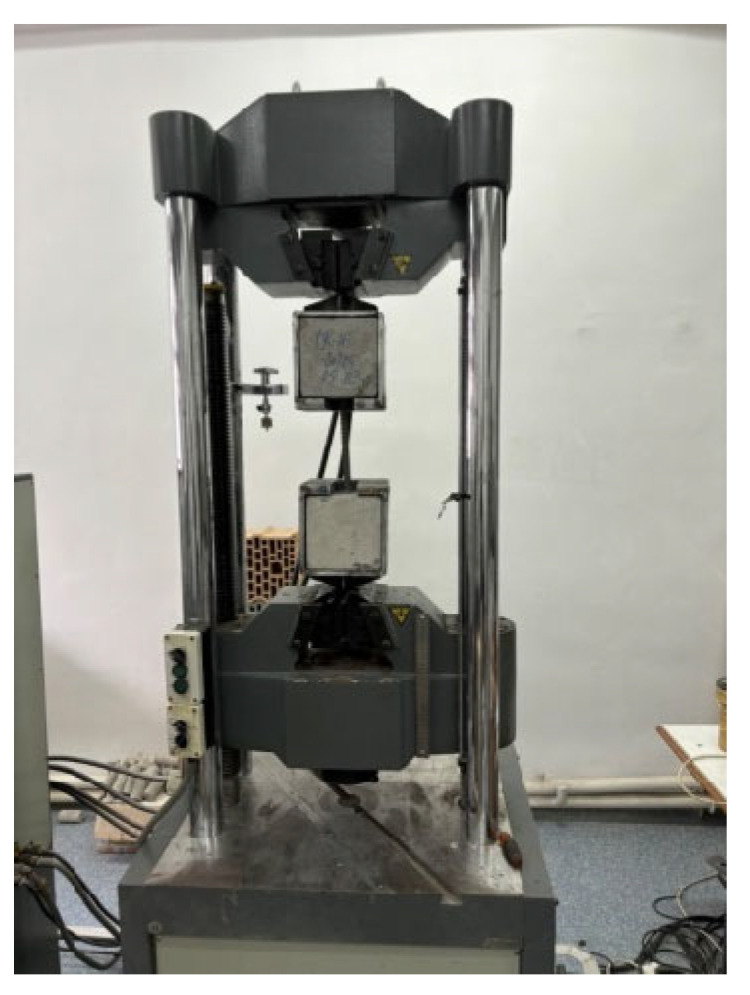
Equipment configuration for the pull-out test.

The tests were carried out in the laboratory using a servo-controlled type WAW 600E electro-hydraulic press (Jinan Testing Equipment IE Corporation, Jinan, China), classified as a universal testing machine with a maximum capacity of 600 kN (Figure 1). This equipment is designed for the mechanical testing of metallic and non-metallic materials under tension, compression, bending, or shear. The force measurement accuracy is ±1%, starting from 20% of the measurement scale. The device allows load control in both displacement- and force-control modes and is equipped with acquisition channels for multiple parameters, including displacement, force, strain, and stress. Loading was applied quasi-statically, at a low pulling rate, until failure occurred in the tested specimens.

**Figure 2:**
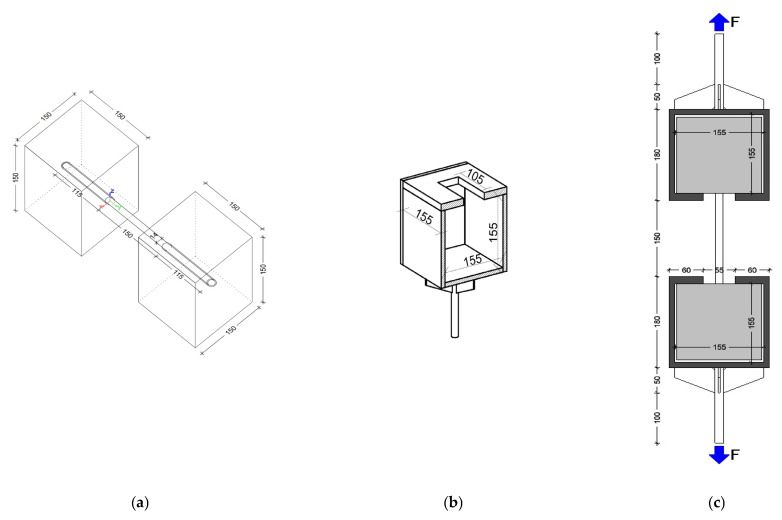
Experimental test sample: (**a**) steel bar fixed in concrete cubes; (**b**) steel casings; (**c**) longitudinal cross-section of the test setup.

To simulate the structural connection between two elements (one existing and one new), an innovative experimental configuration was adopted, in which the reinforcing bar was simultaneously embedded at both ends in concrete cubes with 150 mm edges (Figure 2a). Given the cubic geometry of the specimen ends, which did not allow direct clamping in the testing machine grips, metallic casings made of approximately 5 mm thick steel plates were fabricated and dimensionally adjusted to enclose the concrete cubes. These casings included fastening components enabling attachment to the hydraulic testing equipment (Figure 2b,c). The sole role of these steel casings was to transfer the tensile forces from the testing machine into the concrete cubes, without inducing any additional confinement or altering the stress state at the steel–concrete embedment interface.

In the research presented here, our objective was to reproduce, as faithfully as possible, a realistic coupling scenario in which a steel bar (Ø14 mm) provides the connection between two concrete elements—one old and one new—permanently anchored at both ends. Thus, the bar was simultaneously embedded in two distinct cubes, each symbolizing a structural element.

The concrete cubes were cast in standardized molds, stored, and cured under normal conditions for the standard 28-day maturation period. The resulting cubes were then divided into the following two groups:

- Half of the cubes represented the existing building (R). They were made of concrete with the same strength class (C30/37) and the steel bar was fixed by means of adhesive anchorage;
- The other half represented the proposed new strengthening structure (N). They were made of concrete with different strength classes (C25/30, C30/37, and C35/45). In these cubes, the steel bar was fixed in two alternative configurations, either by adhesive anchorage (with a bonding agent) or monolithic embedment.

In cases where the joint type was monolithic embedment, the steel bar was placed in the fresh concrete and held in position until the concrete matured. In accordance with the manufacturer’s specifications, the adhesive anchorage type was used after the concrete had cured (holes with Ø18 mm diameter and 115 mm depth) by injecting the adhesive and inserting the steel bar. In this way (as illustrated in Figure 2a), several types of specimens/experimental configurations were produced.

In defining the specimens, both the combinations of concrete strength classes between the two cubes (R and N) and the type of anchorage used for the steel bar in each case were taken into account. Thus, six experimental configurations were established, conventionally designated as A, B, C, D, E, and F.

Carrying out the experimental tests for these configurations required specific technical operations. After placing each cube into a metallic casing (Figure 2b), the resulting assemblies were coaxially positioned in the testing machine, with the steel bar aligned along the direction of the force applied by the hydraulic piston. The cube designated as the existing structure was fixed in the lower grip of the testing machine, while that representing the new structure was fixed in the upper grip.

During the test, the applied force acted on the concrete cubes in opposite directions, subjecting the bar to pull-out from both anchorage zones simultaneously. The physical configuration of the specimens during testing is illustrated in Figure 1.

### 2.2. Physical–Mechanical Properties of the Materials Used in Joint Assembly Fabrication

The concrete specimens used in the experiment were produced in compliance with the Romanian technical regulations for force, according to standards [5,20], which establish the requirements for concrete production and monolithic concrete works. Specimen casting was carried out at a certified concrete plant, following standard procedures for preparation, casting, and curing.

The specimens had a cubic geometry, with dimensions of 150 × 150 × 150 mm, and the concrete strength classes were varied between C30/37, C25/30, and C35/45. This range of concrete strength classes was selected to enable a comparative analysis of anchorage performance as a function of concrete strength, reflecting practical scenarios where the existing structure and the new exoskeleton may have different concrete grades. This approach was dictated by the need for a comparative analysis of bar embedment performance as a function of concrete strength class. The concrete mix was designed and produced in accordance with [21], ensuring mixture uniformity and the required quality for testing.

The BST500S type steel bar used in the tests has a nominal diameter of Ø14 mm, nominal yield strength of approximately 500 N/mm^2^ and ultimate tensile strength of ~550 N/mm^2^. The bar length (~380 mm) was chosen so that about 115 mm could be embedded in each concrete cube at the ends.

For the adhesive-anchor cases, a two-component epoxy resin system (Sika AnchorFix-2+) was used; according to the manufacturer’s specifications, for a Ø14 mm rebar, the drilled hole should be 18 mm in diameter and ~115 mm deep, which corresponds to an embedment length of about 8–9 bar diameters to ensure full bond development.

Installation followed the standard procedure: each hole was drilled at the center of the cube and then cleaned (using mechanical brushing and air blast), slightly moistened, and filled with the epoxy adhesive before the steel bar was inserted; subsequently, before testing, the assembly was left to cure for at least 24 h in accordance with the manufacturer’s instructions.

For each concrete class, specimens were prepared to allow the creation of a series combining concrete and anchorage types. In total, six experimental configurations were defined, conventionally designated A–F.

Each configuration consisted of two concrete cubes connected by a single steel bar: one representing the existing structural element (R), and the other the newly added one (N). In order to capture the influence of concrete strength on bond performance, both homogeneous (with the same concrete class in both cubes, C30/37) and heterogeneous configurations (e.g., C30/37 with C25/30 or C30/37 with C35/45) were produced. In addition, for each combination of concrete classes, both anchorage variants—monolithic and adhesive—were tested.

Table 1 presents details regarding the specimens tested in the laboratory, as well as characteristic compressive strength and elastic modulus values for the concrete classes used in cube fabrication.

Configurations A, C, and E featured bilateral (symmetric) adhesive anchorage at both bar ends, whereas configurations B, D, and F were mixed (asymmetric), with one end cast monolithically and the other post-installed with adhesive. We tested both homogeneous (where the ‘new’ and ‘existing’ concrete cubes had the same strength class, e.g., C30/37–C30/37 in Type A) and heterogeneous cases (different concrete classes in the two cubes, e.g., C30/37–C25/30 in Type C or C30/37–C35/45 in Type E). This specimen matrix allowed us to examine the influence of the new concrete’s strength class on the pull-out behavior for each anchorage type. Additionally, for each concrete class combination, both anchorage methods were represented, enabling a direct performance comparison between monolithic embedment and adhesive bonding.

For each experimental configuration defined (A–F), five individual tests were carried out, each using distinct specimens and new reinforcing bars under identical experimental conditions. This approach allowed us to acquire a reproducible and statistically relevant dataset.

In the results processing stage, the obtained values were subjected to an averaging analysis, with extreme values potentially influenced by accidental errors or deviations from standard conditions being discarded. Thus, for each tested configuration, representative mean values were determined for the pull-out force (the maximum tensile force at which the slip of the steel bar was observed in one of the cubes, corresponding to the pull-out moment from the embedment zone) and for the corresponding maximum displacement, expressed as the increase in distance between the two gripping jaws of the specimens in the testing equipment.

### 2.3. Experimental Test Results

The experimental results showed dispersed values: some specimens exhibited significantly higher or lower strengths or deformabilities compared to the mean. To obtain representative values for both force and displacement, these extreme values were excluded.

For instance, with identical concrete on both sides (C30/37 in both cubes), the specimen with adhesive anchors at both ends (Type A) reached a pull-out force of 28.6 kN, whereas the mixed-anchor specimen (Type B, one adhesive end and one cast-in end) achieved 23.4 kN, marking an approximately 18% lower capacity.

**Figure 3:**
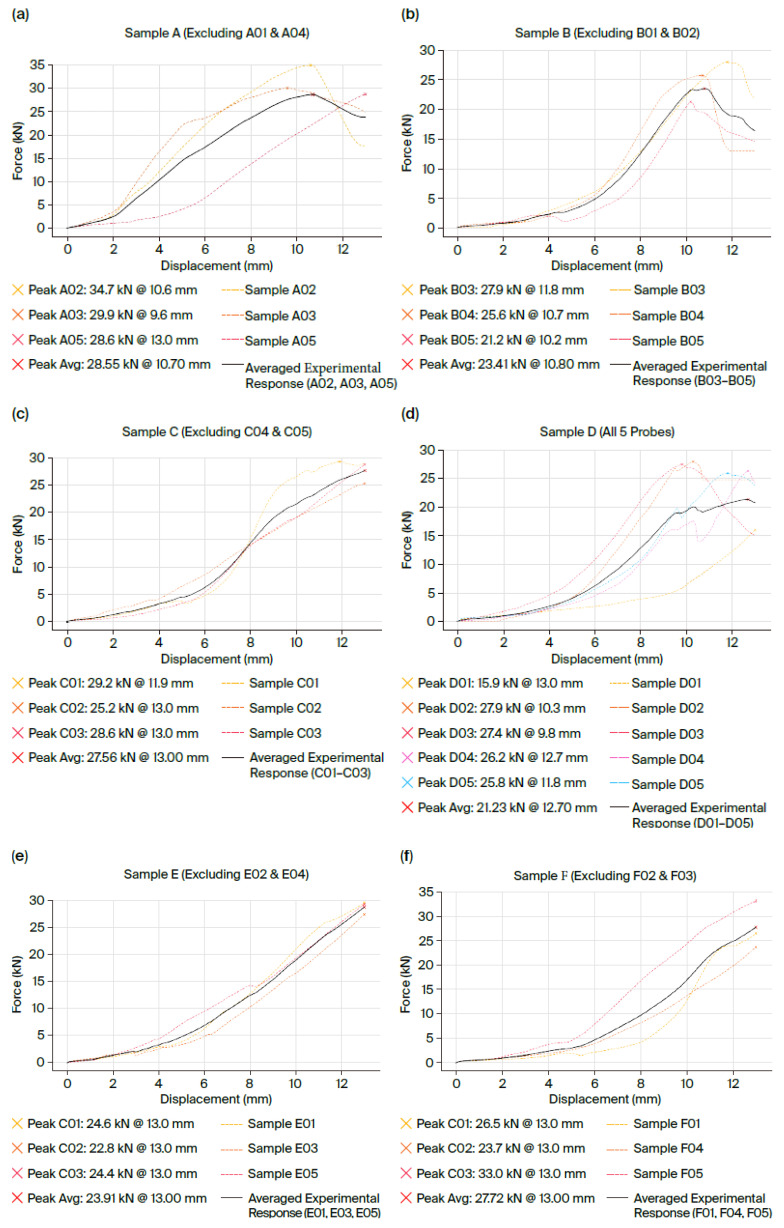
Graphical representation of sample behavior during testing: force–displacement curves for (**a**) samples A; (**b**) B; (**c**) C; (**d**) D; (**e**) E; and (**f**) F.

The graphs in Figure 3 illustrate the force–displacement curves obtained during the tests for each specimen after excluding extreme values, as well as the median curve derived as the arithmetic mean of the force values over the common displacement domain for each test. For each configuration (A-F), five samples were tested. Of these, the curves from the samples that displayed atypical behavior, namely premature failure, were eliminated. As such, these are not presented in the graphs in Figure 3, respectively, for cases (a), (b), (c), (d), (e), and (f). Figure 3 presents the curves obtained from the experimental tests for each configuration, with the selected test curves being those with corresponding behavior.

Table 2 summarizes the mean values obtained from the tests for each specimen, presenting both the maximum pull-out force recorded and the maximum bar displacement (slip) measured at the moment of failure. These values are highlighted in Figure 3.

### 2.4. Interpretation of the Experimental Results

In all analyzed cases, the observed failure mechanism was similar: fracture occurred in the concrete at the steel bar’s contact zone, with or without adhesive, without the development of plastic deformations in the reinforcement. This behavior indicates that the pull-out resistance was governed by the tensile and shear capacity of the concrete in the specimen. The mechanical properties of the adhesive or the steel did not visibly influence the specimens’ behavior under pull-out loading. This concrete-governed failure mode is consistent with observations in other steel–concrete connector systems. Pull-out tests on the samples showed that ultimate capacity is dictated by concrete cracking and shear failure around the connector, rather than steel component yielding [22,23].

**Figure 4:**
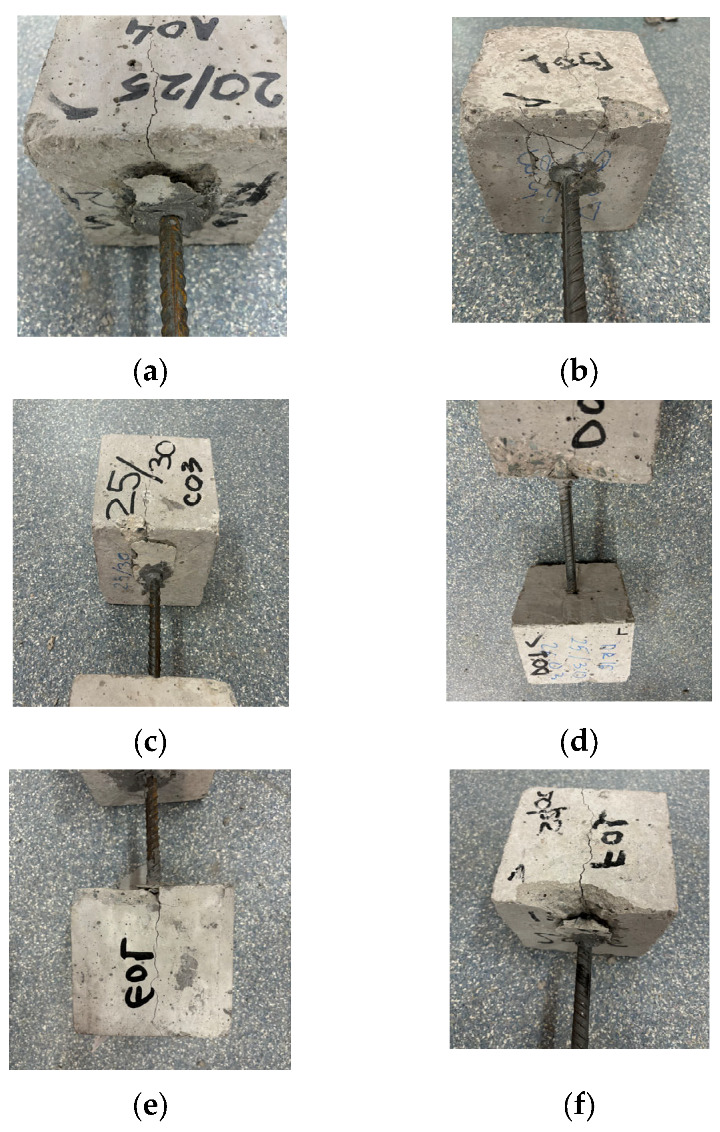
Failure modes in experimental tests: (**a**) samples A; (**b**) B; (**c**) C; (**d**) D; (**e**) E; and (**f**) F.

Figure 4 highlights the failure phenomenon, which manifested through pronounced cracking and local concrete spalling in the bar exit zone—a behavior specific to pull-out failure—in the case of both direct bar embedment and adhesive anchorage.

In all specimens, within the cube where failure initiation and bar pull-out occurred, visible cracks and fractures developed along the cube’s height. These cracks were caused by tensile stresses in the concrete, oriented perpendicular to the loading direction. It should also be noted that the assumed homogeneity of concrete is false, as it is a heterogeneous material that may contain numerous large pores or voids. Therefore, it is reasonable to assume that the failure of the loaded specimen may also be governed by its shear envelope parameters, namely cohesion and internal friction angle (*c*, *ϕ*). Consequently, the behavior under an applied load is also related to internal shear failure [24].

An analysis of the experimental test results highlights that the tensile and shear strengths of the concrete were exceeded, leading to failure and bar pull-out. All the tests indicated that the concrete represented the weak link of the system, while the steel bar did not exhibit any visible deformations or damage.

**Figure 5:**
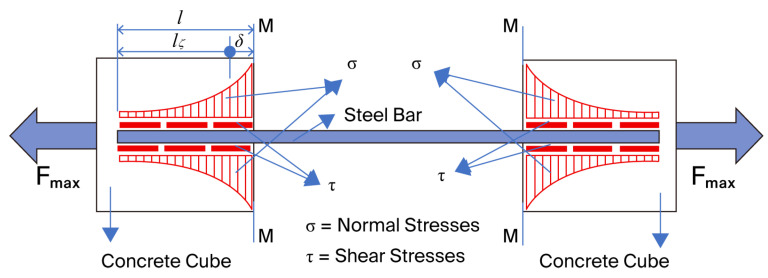
Stress states in the tested samples: diagram of normal and shear stresses.

The behavior of the specimens during testing highlights the occurrence of normal tensile stresses, *σ*, as well as shear stresses, *τ*, as illustrated in Figure 5.

Based on the details shown in Figure 5, this failure mechanism can be demonstrated through an analytical calculation model, as presented in the following section.

Regulation [8] specifies how the characteristic shear strength can be approximated, given the characteristic cube compressive strength, using the following relation (1):

(1) $$f_{vk}=\sqrt{0.8\cdot f_{ck,cube}}$$

The bond between two materials is destroyed when the shear capacity of the weaker material in the joint is exceeded. In our specimens, the bond between the steel bar and the adhesive used for anchorage was much stronger than that between the bar or adhesive and the concrete. Therefore, the bond surface governing the pull-out tests was the interface in contact with the concrete of the cube.

The distribution area of the bar pull-out force in each cube—at the bond surface be-tween the concrete and the steel bar or the adhesive encasing it, *A_f_*—can be calculated using relation (2), where the embedment length, *l*, is 115 mm and the diameter *Ø* is 14 mm for the bar embedded in concrete, or 18 mm for that anchored with adhesive:

(2) $$A_{f}=\pi \cdot \emptyset \cdot l=361.1\cdot \emptyset$$

The shear stress at the interface between the concrete and the steel bar or the adhesive is calculated using relation (3):

(3) $$\tau =F_{max}/\left(A_{f}^{R}+A_{f}^{N}\right)$$

Table 3 presents the values of the characteristic shear strength of concrete, calculated according to Equation (1), as well as the values of the shear stresses, *τ*, evaluated according to Equation (3), which develop when the pull-out force reaches its maximum value, *F_max_*, obtained for each specimen in the experimental tests.

An analysis of the experimental results revealed that the zero-bond phenomenon occurred between the bar—or its adhesive—and the concrete. This phenomenon is initiated by the development of tensile stresses in the concrete, oriented perpendicular to the loading direction. These stresses lead to the formation of microcracks in the concrete which, as the applied force increases, coalesce and propagate toward the bar end (Figure 4). Thus, the zero-bond condition begins to manifest over a length, *δ* ([25]) starting from section M–M (Figure 5), in each cube. Considering the characteristic shear strengths that vary for different concrete strength classes, as well as the varying hole diameters in the cubes for bar anchorage, the length of the zero-bond zone, *δ*, has an average value that can be estimated using relation (4):

(4) $$\delta =l-\left(F_{max}/(\pi \cdot (\emptyset_{R}\cdot f_{vk}^{R}+\emptyset_{N}\cdot f_{vk}^{N})\right)$$

where

– $\emptyset_{R}$ and $\emptyset_{N}$ are the diameters of the drilled hole (core) for the bar or the bar with adhesive, specific to each cube, R or N;
– $f_{vk}^{R}$ and $f_{vk}^{N}$ are the characteristic shear strengths of the concrete in cubes R and N, respectively.

In Table 3, the mean values of *δ* are evaluated using relation (4) for the maximum force equal to the mean value obtained from the experimental tests, the diameter of the drilled hole (core) in the concrete, and the shear strengths of the concrete calculated with relation (1). A post-test analysis of the specimens revealed the following:

(a) When the maximum value of *δ* is reached, the embedment length in the concrete is reduced to a critical length, *l_crit_ = l* − *δ*, and the shear stress values exceed the shear strengths of the concrete at the interface between the concrete and the bar, or between the concrete and the bar with adhesive.
(b) The exceedance of shear strengths is influenced by the length of the zero-bond zone, *δ*, the diameter of the anchorage hole of the bar in the concrete, and the concrete strength class.
(c) In the situation where the concrete strength class of the two cubes is identical and the diameter of the anchorage hole of the bar is the same, the length of the zero-bond zone, *δ*, is similar for both cubes, and the value of the critical embedment length can be approximated as the value estimated with Equation (5):

(5) $$l_{crit}=l-\delta$$

(d) In the situation where the concrete strength classes of the two cubes are different and the diameter of the anchorage hole of the bar is the same, the critical length for the two cubes is different and is affected by a coefficient *C_i_*, which is proportional to the ratio between the characteristic shear strengths. The critical embedment length for each end can be evaluated using relations (6) and (7):

(6) $$l_{crit}^{R}=C_{i}\cdot \left(l-\delta \right)=\frac{f_{vk}^{R}}{f_{vk}^{N}}\cdot \left(l-\delta \right)$$

(7) $$l_{crit}^{N}=C_{i}\cdot \left(l-\delta \right)=\frac{f_{vk}^{N}}{f_{vk}^{R}}\cdot \left(l-\delta \right)$$

(e) In the situation where the concrete strength classes of the two cubes are not identical and the diameter of the anchorage hole of the bar is different (due to the presence of adhesive at only one bar end), the critical embedment length for the two cubes is different and is affected by a coefficient *C_i_*, which is proportional to the product of the ratios between the characteristic shear strengths and the diameters. The critical embedment length for each end can be evaluated using relations (8) and (9):

(8) $$l_{crit}^{R}=C_{i}\cdot \left(l-\delta \right)=\frac{f_{vk}^{R}}{f_{vk}^{N}}\cdot \frac{\emptyset^{R}}{\emptyset^{N}}\left(l-\delta \right)$$

(9) $$l_{crit}^{N}=C_{i}\cdot \left(l-\delta \right)=\frac{f_{vk}^{N}}{f_{vk}^{R}}\cdot \frac{\emptyset^{N}}{\emptyset^{R}}\left(l-\delta \right)$$

(f) The length of the zero-bond zone, *δ*, for all cases is estimated using relation (4).

In all cases, total failure is initiated and bar pull-out occurs (with or without adhesive, depending on the anchorage type) through the exceedance of shear strength along the reduced embedment length. The pull-out is accompanied by bar slip. The shear stresses along the reduced embedment length are calculated using relations (10) and (11), with the results summarized in Table 4.

(10) $$\tau^{R}=F_{max}/(2\cdot \pi \cdot (l_{crit}^{R}\cdot \emptyset^{R})$$

(11) $$\tau^{N}=F_{max}/(2\cdot \pi \cdot (l_{crit}^{N}\cdot \emptyset^{N})$$

In the experimental tests, it was observed that the pull-out force of the bar, with or without adhesive (depending on the anchorage type), *F_max_*, as well as the maximum displacement, Δ*_max_*, were influenced by several factors:

– The bond between the concrete and the steel bar, or the adhesive used for bar anchorage;
– The confinement effect resulting from concrete drying shrinkage;
– The effect of concrete quality and strength on tension and compression, as well as its degree of homogeneity;
– The mechanical anchorage effect of the bar ends in the concrete cube through the embedment length;
– The diameter of the bar, with or without adhesive, as it influences the development of cracks at zero-bond phenomenon onset.

The individual contributions of these factors are difficult to separate or quantify and it is therefore challenging to compare and draw definitive conclusions regarding specimen behavior. An analysis of the data in Table 4 highlights the following aspects:

– For type A specimens, failure was initiated in either of the two cubes, and it is reasonable to assume that bar pull-out occurred in the cube where structural defects existed between the adhesive and the concrete in the reduced bond zone, caused by a non-uniform distribution of the former. These structural defects locally disturbed the stress state through a stress concentration phenomenon.
– For type B specimens, failure was initiated in the cube where the steel bar was monolithically embedded (without adhesive):

$\tau^{N}$ = 8.42 MPa > $f_{vk}^{N}$ = 5.73 MPa.

– For type C specimens, even though anchorage was symmetrically executed with adhesive, failure was initiated in the cube with the lower strength class:

$\tau^{N}\;$= 6.00 MPa > $f_{vk}^{N}$ = 5.23 MPa.

– For type D specimens, failure was initiated in the cube with the lower strength class, where the steel bar was monolithically embedded (without adhesive):

$\tau^{N}\;$= 8.87 MPa > $f_{vk}^{N}$ = 5.23 MPa.

– For type E specimens, even though anchorage was symmetrically executed with adhesive, failure was initiated in the cube with the lower strength class:

$\tau^{R}\;$= 6.68 MPa > $f_{vk}^{R}$ = 5.73 MPa.

– For type F specimens, failure was initiated in the cube with the higher strength class; however, in this case, the steel bar was monolithically embedded (without adhesive):

$\tau^{N}\;$= 7.96 MPa > $f_{vk}^{N}$ = 6.34 MPa.

In conclusion, it can be stated that anchorage type—either adhesive or monolithic—and concrete quality can be considered major factors influencing pull-out behavior in the experimental study presented.

## 3. FEM Analysis of the Pull-Out Behavior of the Structural Anchorage Systems Studied

### 3.1. Numerical Modeling of the Pull-Out Response of the Concrete–Reinforcement Connection in the Context of Structural Strengthening

Numerical modeling was carried out in ANSYS Workbench 2022 R2, a well-established finite element analysis software with extensive applications in structural engineering. The three-dimensional geometry of the testing system was initially developed in Autodesk Inventor 2025, accurately reflecting the dimensions and configuration of the experimental assembly analyzed in the case study. The geometric model was then imported into the ANSYS platform, where the mechanical properties of the materials were defined based on the values previously determined in the experimental stage, thereby ensuring consistency between numerical modeling and actual testing conditions.

For the numerical simulation, an FEA model was developed within the Explicit Dynamics module of ANSYS Workbench 2022 R2, with the objective of analyzing the local behavior of the concrete–anchor system subjected to an axial load applied to the reinforcing bar. The modeling specifically targeted the complex interaction between the concrete, adhesive, and reinforcing steel, focusing on the shear stress transfer responsible for initiating the pull-out phenomenon. The choice of the explicit method allowed for an accurate representation of the nonlinear material behavior, including the progressive degradation of the concrete and the occurrence of sudden failures [26].

**Figure 6:**
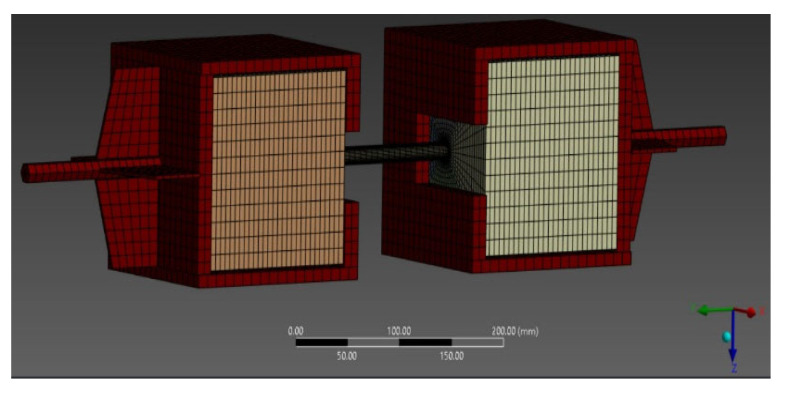
Geometric discretization of the 3D finite element model for samples.

The domain was discretized using a refined mesh of solid finite elements, consisting of 76,589 nodes and 65,676 elements, a configuration considered suitable for capturing local stress distribution and representing concrete crack propagation under axial loading. This resolution (Figure 6) enables a detailed representation of stress concentration zones, particularly in critical regions around anchors and at material interfaces.

Within the Explicit Dynamics module, the specific physical–mechanical properties of each material involved were defined: the concrete of varying strength classes, the adhesive compound (adhesive anchor), and the reinforcing steel. The parameter values were established in correlation with the data obtained from the experimental program, as well as with theoretical models previously validated in the literature.

For the adhesive used, Sika AnchorFix-2+, the characteristic compressive strength value of 68 MPa was introduced into the model, in accordance with the specifications provided in the manufacturer’s official technical documentation. This value reflects the capacity of the epoxy adhesive to sustain high stresses, making it suitable for demanding structural applications, including seismic conditions or long-term exposure.

To define the behavior of the reinforcing bars in the numerical model, we adopted the isotropic properties specific to structural steel commonly used in construction engineering. The input parameters were longitudinal elastic modulus *E* = 210,000 MPa, tangent modulus *G* = 1500 MPa, Poisson’s ratio *ν* = 0.3, density *ρ* = 7800 kg/m^3^, and yield strength *f_y_* = 450 MPa. This formulation allows for the representation of the linear–elastic behavior of steel up to the yielding threshold, in accordance with the requirements specified in [27].

The values for concrete material used in FE models are presented in Table 5. The characteristic compressive strengths and elasticity modulus values, *f_ck,cube_* and *E*, given in Table 1, were introduced into the model, along with the values evaluated using relations from [28], as given in Table 5.

In addition, to complete the dataset required for material definition, we employed standardized relations from the literature and technical codes to estimate the secondary properties of the modeled materials. These parameters were essential for realistically simulating material transition from the elastic to plastic domain, as well as for accurately reproducing the mechanisms of progressive cracking and pull-out observed in the experimental tests.

The nonlinear behavior of concrete under dynamic loading was simulated using the constitutive model developed by [29], a model that was later extended and refined by [30], and validated in subsequent research on impact and penetration in reinforced concrete [31]. This model provides an advanced representation of degradation, cracking, and local failure processes, making it suitable for simulating bar pull-out under realistic loading conditions.

To simulate the pull-out behavior of the bar, it was assumed that the concrete was in a cracked state at the moment the pull-out force was applied. Consequently, the elastic modulus was reduced to approximately half of the value corresponding to uncracked concrete. This assumption reflects the influence of microcracks on the global stiffness of the concrete element during loading.

The choice of the constitutive model proposed by Riedel was based on its proven ability to reproduce complex dynamic behaviors, such as the formation of the cracking cone, stress redistribution within the concrete mass, and gradual bond loss at the concrete–anchor interface. These phenomena observed during the experimental tests could be accurately captured in the numerical simulations due to the nonlinear and time-dependent nature of the model implemented.

### 3.2. Investigation of Stress and Strain Distribution in the Joint Models

Within the Explicit Dynamics module of ANSYS Workbench 2022 R2, each geometric entity was assigned distinct material properties, in accordance with the physical–mechanical characteristics of the materials used in the experimental model. In configurations with adhesive anchors, both the parameters specific to concrete and those corresponding to the composite adhesive were introduced in order to realistically reproduce the interaction between the two media; this is essential for accurately simulating stress transfer at the interface.

For the contact zones resulting from casting concrete around the reinforcement or the anchor—namely the concrete–reinforcement and concrete–adhesive anchor interfaces—bonded contact conditions were applied, assuming a full connection between surfaces with no relative slip. This contact assumption simulates a complete transfer of bond stresses, making it suitable for analyzing behavior under pull-out loading.

For the interface between the concrete mass and the specimen’s metallic casing, we applied a frictionless contact condition, allowing free sliding between surfaces without resistance to tangential displacement. This approach more accurately reflects the real behavior of independent components, without mechanical connection or adhesion between them, under the testing conditions.

**Figure 7:**
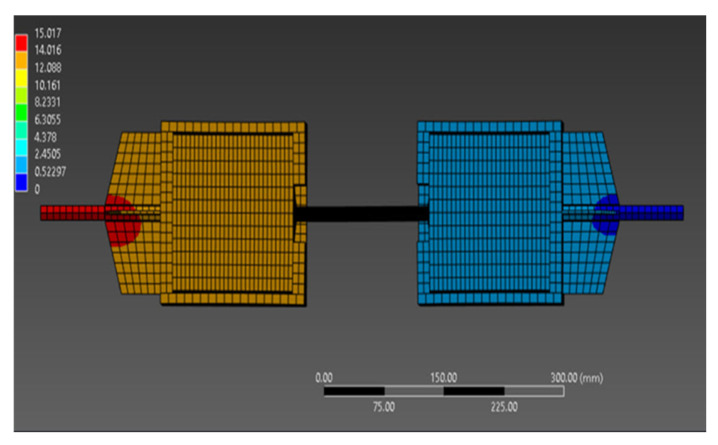
Test highlighting sample finite element modeling.

The finite element analysis results are graphically illustrated in Figure 7 and summarized in Table 6, highlighting the maximum force values and corresponding displacements for each tested configuration (A–F).

Characterized by bilateral adhesive anchorage, configurations A, C, and E exhibited the highest load-bearing capacities. In contrast, configurations B, D, and F with mixed anchorage arrangements (adhesive at one end and monolithic at the other) recorded failures at lower force levels and smaller associated deformations, indicating inferior performance compared to those with fully adhesive anchorage.

## 4. Comparative Analysis Between Numerical Simulations and Experimental Tests on Anchorage Systems: A Discussion

**Figure 8:**
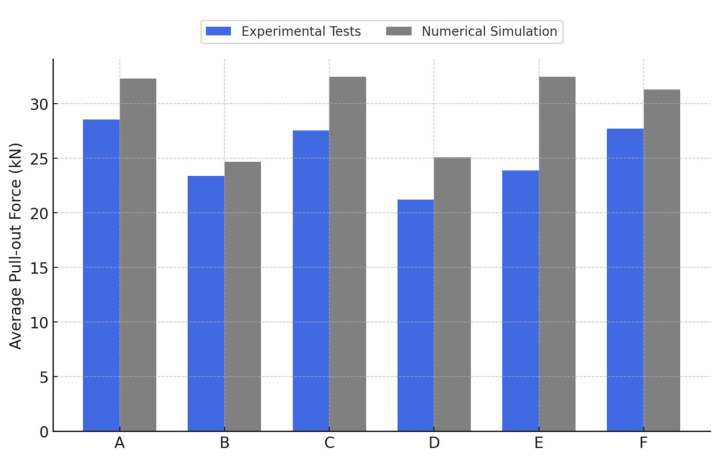
Comparative study on the mean pull-out resistance values obtained experimentally and through numerical modeling for concrete–steel joints.

**Figure 9:**
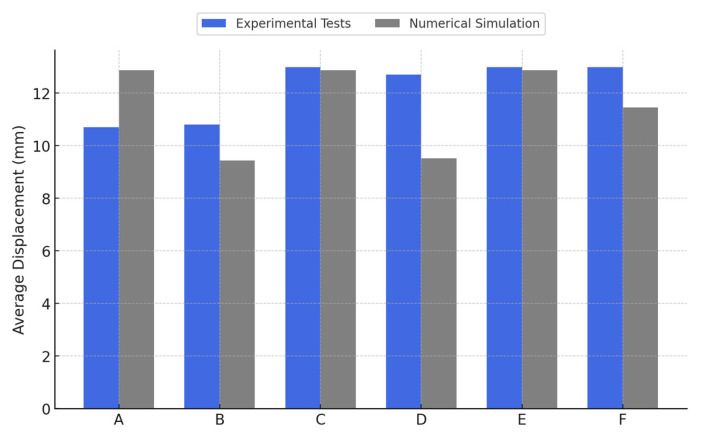
Comparative study of the mean deformational response under pull-out loading: experimental data versus computational modeling.

An analysis comparing the results obtained through numerical simulation in ANSYS and those from the experimental tests revealed a significant correlation, both in terms of the maximum pull-out force values and the observed failure modes. This agreement, as graphically illustrated in Figure 8 and Figure 9, validates the accuracy of the adopted model and supports the applicability of the numerical method for evaluating concrete–steel connection behavior.

In our experiments, for configurations with bilateral adhesive anchorage (A, C, E) we generally recorded values comparable to—or slightly higher than—those obtained in mixed configurations (B, D, F). For example, in homogeneous C30/37 concrete, configuration A (bilateral adhesive anchorage) developed an average force of 28.6 kN, while configuration B (one monolithic and one adhesive end) reached 23.4 kN, marking a difference of approximately 18%.

Furthermore, no clear increasing trend in pull-out capacity was observed with the higher strength of the ‘new’ concrete cube for the bilateral adhesive-anchored specimens. Among the fully adhesive cases, the peak loads were 28.6 kN for specimen A (C30/37–C30/37), 27.6 kN for specimen C (C30/37–C25/30), and 23.9 kN for specimen E (C30/37–C35/45). This deviation from the initially expected trend suggests that execution quality and local interface conditions (at the bar–adhesive bond) may play a decisive role in the overall performance, sometimes overshadowing the effect of concrete strength class on the new element.

By contrast, for the mixed anchorage specimens, we saw a more consistent influence from the new concrete’s strength: using a higher concrete class on the new side tended to increase the pull-out capacity. Specifically, specimen B (with new concrete C30/37) developed 23.4 kN and specimen D (new concrete C25/30) reached 21.2 kN, while specimen F (new concrete C35/45) attained 27.7 kN. Notably, the strength achieved by specimen F (with the highest new-concrete class) was comparable to that of the fully adhesive specimens, suggesting that upgrading the concrete quality in the new element can partially compensate for having only one end adhesively anchored.

In terms of ductility (slip at failure), both anchorage systems exhibited very similar performances: the fully adhesive-anchored specimens (A, C, E) showed failure displacements in the range of roughly 10.7 mm to 13.0 mm, while the mixed-anchor specimens (B, D, F) had ultimate displacements between about 10.8 mm and 13.0 mm.

The finite element simulations tended to overestimate the pull-out strength compared to the experiments: for bilateral adhesive cases, FEA predicted peak forces of about 32.3–32.5 kN versus 23.9–28.6 kN, while mixed cases saw 24.7–31.3 kN versus 21.2–27.7 kN. These differences (on the order of 10–20% higher in the model) can be attributed to the difficulty of accurately reproducing the initial bond conditions of the cast-in (monolithic) end in the model, as well as to the inherent variability in the manual casting/anchoring process during experiments.

With regard to displacement, the numerical model’s predictions were generally in good agreement with the tests (predicted failure displacements ranged between 9.4 mm and 12.9 mm, comparable to the experimental 10–13 mm range); however, one notable discrepancy was for specimen F, where the simulation gave a lower failure displacement (11.45 mm) compared to the 13.0 mm observed experimentally, indicating the model’s sensitivity to bar–concrete contact representation and material parameters for that configuration.

Numerical modeling provided additional insight into the behavior of the anchorage system, showing that a fully adhesive (bilateral) anchorage produces a more uniform stress distribution along the bar, contributing to higher peak forces and a more gradual, ductile load–displacement response. The mixed anchorage configuration, on the other hand, led to stress concentrations and earlier bond failure.

Moreover, the FE simulations confirmed that failure initiated at the concrete–adhesive interface via the formation of a characteristic concrete pull-out cone and cracking in the surrounding concrete, while the steel bar remained elastic—exactly as observed in the experiments—thereby validating the assumptions used in the model.

Validating the numerical models through correlation with experimental data provides a solid methodological basis for extending the analysis at a parametric level, as well as for applying the results to real structural scenarios specific to strengthening projects with reinforced concrete exoskeletons. Within this framework, finite element analysis is not limited to a predictive function but can be employed as a technical decision-support tool, contributing to intervention solution optimization for existing buildings.

The numerical models developed in the Explicit Dynamics module accurately reproduced the global behavior of the tested system, highlighting the concentration of shear stresses at the bar–adhesive anchor interface and the formation of the pull-out cone around the anchorage point, a phenomenon that was also observed in the experimental tests.

For configurations with adhesive anchorage at both ends (types A, C, E), the values obtained through numerical simulation significantly exceeded the experimental results, with percentage differences ranging between 13 and 36%. For mixed configurations (types B, D, F), the results showed more moderate deviations, between 5% and 18%, which can be attributed both to the difficulty of faithfully reproducing the bond conditions of the monolithic end and to the inherent variations in the experimental casting and anchoring process.

The strain distribution in the simulated models indicated the more ductile behavior of joints with bilateral adhesive anchorage, as was similarly observed in the experimental program. In all analyzed cases, no steel bar fracture was recorded, and failure occurred exclusively through bond loss at the concrete–adhesive interface. These results confirm the hypothesis that, in terms of the load-bearing capacity of the tested joint system, concrete represents the limiting element.

This close agreement between the numerical and experimental results provides a robust basis for extending the analysis to parametric studies and applying the model to practical design scenarios. Essentially, the validated FE model can serve as a decision-support tool for optimizing anchorage configurations in real strengthening projects involving concrete exoskeletons.

## 5. Conclusions

This paper proposes and validates an original experimental approach consisting of testing the connections between two concrete elements by means of Ø14 mm bars, anchored either with adhesive or monolithically in the concrete anchorage zones. This configuration accurately reproduces a real structural strengthening intervention situation, where efficient connection between existing and added structures is required. The absence of direct documented studies on this type of test justifies the relevance of the experimental approach, highlighting its innovative character and its contribution to the field of adhesive anchor performance evaluation in real applications.

Our analysis of the experimental results confirms that the anchorage method directly influences the pull-out behavior of reinforcing bars. The results indicate that symmetrical adhesive anchorage allows a more uniform distribution of stresses at the bar–concrete interface, leading to a higher load-bearing capacity compared to mixed solutions. The deviations from the initial hypothesis suggests that execution quality and local conditions at the bar–adhesive interface may play a decisive role in the final performance.

Notably, the choice of anchorage type did not significantly affect the overall deformation capacity (ductility) of the connection; both fully adhesive and mixed-anchor specimens exhibited similar failure displacements. However, fully adhesive (bilateral) anchorage tended to show a slightly more gradual bond failure, indicating a more ductile process compared to the more abrupt bond loss for mixed (monolithic) anchorage.

The numerical simulations largely confirmed the trends observed in the experiments, although pull-out force values were slightly overestimated for most configurations.

Overall, both the experimental and numerical data confirm that bilateral adhesive anchorage provides a more stable and predictable performance compared to mixed configurations. At the same time, the class of the new concrete influences the behavior differently, highlighting the importance of proper execution and detailed material characterization in the design and analysis process.

Examining the tested specimens, we found that all observed failures occurred within the concrete mass; no fracture or plastic deformation was observed in the steel bar. The deformations manifested as the cracks and cone-shaped failures typical of axial pull-out loading.

Our experimental results confirm the effectiveness of using adhesive anchors for creating connections between external strengthening elements, such as exoskeletons, and existing structures. Under certain application conditions, this anchoring method even proved superior to traditional solutions based on monolithic reinforcement continuity. The tests also validated the use of the double-embedment experimental configuration as an alternative method for evaluating pull-out behavior, despite the fact that this approach is not explicitly regulated in current testing standards. The information obtained from these tests provides a solid basis for developing subsequent numerical models (finite element analyses) to optimize structural strengthening solutions in seismic contexts.

Building on these findings, future studies should investigate other parameters and conditions to broaden the applicability of the results. For instance, additional pull-out tests with different bar diameters or embedment depths, as well as experiments using varying adhesive types or aging/sustained loads, would help generalize the conclusions. Likewise, expanded parametric finite element analyses (e.g., exploring different concrete strength combinations, cyclic loading, or larger-scale anchorages) are recommended to further optimize anchor design and develop improved guidelines for structural strengthening applications.

## Figures and Tables

**Table 1:** Characteristics of the experimental samples used in the pull-out behavior analysis.

Sample	Connection System	Type of Embedding		Estimated Concrete Strength Class		Concrete Characteristic Compressive Stress *f_ck,cube_* [MPa]		Concrete Elasticity Modulus *E* [GPa]	
		Steel Bar/Cube R	Steel Bar/ Cube N	Cube R	Cube N	Cube R	Cube N	Cube R	Cube N
A	Symmetric	Adhesive anchorage		C30/37		41.1		33	
			Adhesive anchorage		C30/37		41.1		33
B	Asymmetric	Adhesive anchorage		C30/37		41.1		33	
			Concrete embedment		C30/37		41.1		33
C	Symmetric	Adhesive anchorage		C30/37		41.1		33	
			Adhesive anchorage		C25/30		34.3		31
D	Asymmetric	Adhesive anchorage		C30/37		41.1		33	
			Concrete embedment		C25/30		34.3		31
E	Symmetric	Adhesive anchorage		C30/37		41.1		33	
			Adhesive anchorage		C35/45		50.2		34
F	Asymmetric	Adhesive anchorage		C30/37		41.1		33	
			Concrete embedment		C35/45		50.2		34

**Table 2:** Average values obtained for the maximum force and corresponding maximum displacement in the experimental tests.

Samples	Maximum Test Force *F_max_* [kN]	Maximum Displacement Δ*_max_* [mm]
A	28.55	10.70
B	23.41	10.80
C	27.56	13.00
D	21.23	12.70
E	23.91	13.00
F	27.72	13.00

**Table 3:** Mean values obtained for the characteristic shear strength and perimeter shear stress.

Type	$f_{ck,cube}$ [MPa]		$f_{vk}$ Conf. Equation (1) [MPa]		$F_{max}$ [kN]	τ Conf. Equation (3) [MPa]		δ Conf. Equation (4) [mm]	
	cub R	cub N	cub R	cub N		cub R	cub N	cub R	cub N
A	41.1		5.73		28.55	2.133		70.92	
		41.1		5.73			2.133		70.92
B	41.1		5.73		23.41	1.801		74.34	
		41.1		5.73			2.315		74.34
C	41.1		5.73		27.56	2.402		70.51	
		34.3		5.23			2.402		70.51
D	41.1		5.73		21.23	1.633		76.66	
		34.3		5.23			2.100		76.66
E	41.1		5.73		23.91	1.839		79.95	
		50.2		6.34			1.839		79.95
F	41.1		5.73		27.72	2.132		69.00	
		50.2		6.34			2.742		69.00

**Table 4:** Shear strength values along the reduced embedment length.

Specimen	$f_{vk}$ Conf. Equation (1) [MPa]		$F_{max}$ [kN]	δ Conf. Equation (4) [mm]		$C_{i}$ Conf. Equations (6) and (7)		$l_{crit}$ Conf. Equations (8) and (9) [mm]		τ Conf. Equations (10) and (11) [MPa]	
	cub R	cub N		cub R	cub N	cub R	cub N	cub R	cub N	cub R	cub N
A	5.73		28.55	70.92		1.0000		42.81		5.73	
		5.73			70.92		1.0000		42.81		5.73
B	5.73		23.41	74.34		1.2857		52.28		3.96	
		5.73			74.34		0.7778		31.62		8.42
C	5.73		27.56	70.51		1.0956		55.23		5.00	
		5.23			70.51		0.9127		46.02		6.00
D	5.73		21.23	76.66		1.4086		54.00		3.48	
		5.23			76.66		0.7099		27.22		8.87
E	5.73		23.91	79.95		0.9038		31.68		6.68	
		6.34			79.95		1.1065		38.78		5.45
F	5.73		27.72	69.00		1.1620		53.46		4.58	
		6.34			69.00		0.8606		39.59		7.96

**Table 5:** Concrete properties used in FEM.

Material Properties	Concrete C25/30	Concrete C30/37	Concrete C35/45
Density [kg/m^3^]	2400	2400	2400
Compressive strength, *f_ck,cil_* = 0.8*f_ck,cube_* [MPa]	27.44	32.88	40.16
Tensile strength, *f_ctm_* = 0.3(*f_ck,cil_*)^2/3^ [MPa]	2.73	3.08	3.52
Shear strength *f_vk_* = (*f_ck,cil_*)^1/2^ [MPa]	5.23	5.73	6.34
Poisson’s Ratios	0.2	0.2	0.2
Shear Modulus, *G* = *E*/[2(1 + *ν*)] [MPa]	12,917	13,750	14,167
Minimum Strain to Failure	0.01	0.01	0.01

**Table 6:** Results from FEA simulations of experimental tests.

Samples	Maximum Force *F_max_* [kN]	Maximum Displacement Δ*_max_* [mm]
A	32.30	12.876
B	24.70	9.438
C	32.50	12.876
D	25.10	9.526
E	32.50	12.876
F	31.30	11.450

## Data Availability

The original contributions presented in this study are included in the article. Further inquiries can be directed to the corresponding author.

## Abbreviations

The following abbreviations are used in this manuscript:

ACI	American Concrete Institute
ANSYS	Analysis System (Finite Element Software)
EN	European Norm
ETAG	European Technical Approval Guideline
EOTA	European Organisation for Technical Approval
FEA	Finite Element Analysis
NE	Normativ Execuție
SR	Romanian Standard
TR	Technical Report

## References

[1] Yilmaz A., Aydin E., Arslan G. (2013). Behaviour of post-installed anchors in low strength concrete. Constr. Build. Mater..

[2] Eligehausen R., Cook R.A., Appl J. (2006). Behavior and design of adhesive bonded anchors. ACI Struct. J..

[3] European Organisation for Technical Approvals (EOTA) (2010). Technical Report TR 029: Design of Bonded Anchors.

[4] European Organisation for Technical Approvals (EOTA) (1997). ETAG 001—Guideline for European Technical Approval of Metal Anchors for Use in Concrete.

[5] Romanian Standards Association (ASRO) (2019). SR EN 1992-4:2019—Eurocode 2: Design of Concrete Structures.

[6] Konieczny K., Dudek D., Kukiełka A. (2024). Load capacity of screw anchor installed in concrete substrate reinforced with steel fibers depending on fiber content. Materials.

[7] Spyridis P., Mellios N. (2022). Tensile performance of headed anchors in steel fiber reinforced and conventional concrete in uncracked and cracked state. Materials.

[8] Khan Q.S., Akbar H., Qazi A.U., Kazmi S.M.S., Munir M.J. (2024). Bond stress behavior of a steel reinforcing bar embedded in geopolymer concrete incorporating natural and recycled aggregates. Infrastructures.

[9] Su T., Wang C., Cao F., Zou Z., Wang C., Wang J., Yi H. (2021). An overview of bond behavior of recycled coarse aggregate concrete with steel bar. Rev. Adv. Mater. Sci..

[10] Devaraj R., Olofinjana A., Gerber C. (2023). On the factors that determine the bond behaviour of GFRP bars to concrete: An experimental investigation. Buildings.

[11] Amin M.N., Iqbal M., Althoey F., Khan K., Faraz M.I., Qadir M.G., Alabdullah A.A., Ajwad A. (2022). Investigating the bond strength of FRP rebars in concrete under high temperature using gene-expression programming model. Polymers.

[12] Wohlfahrt D., Peller H.F.M., Müller S., Modler N., Mechtcherine V. (2023). Investigation of helix-pultruded CFRP rebar geometry variants for carbon-reinforced concrete structures. Polymers.

[13] Yoo S.J., Hong S.H., Yoon Y.S. (2023). Bonding behavior and prediction of helically ribbed CFRP bar embedded in ultra high-performance concrete (UHPC). Case Stud. Constr. Mater..

[14] American Concrete Institute (ACI) (2011). ACI 355.4-11—Qualification of Post-Installed Adhesive Anchors in Concrete.

[15] Cook R.A., Doerr G.T., Klingner R.E. (1993). Bond stress model for design of adhesive anchors. ACI Struct. J..

[16] American Concrete Institute (ACI) (2019). ACI 318-19—Building Code Requirements for Structural Concrete.

[17] Darwin D., McCabe S.L. (1993). Development length criteria: Confinement effects and bond mechanics. ACI Struct. J..

[18] Yang Y., Nakamura H., Miura T., Yamamoto Y. (2019). Effect of corrosion-induced crack and corroded rebar shape on bond behavior. Struct. Concr..

[19] Natino M.R.L., Yang Y., Nakamura H., Miura T. (2021). Experimental study on bond behavior of corroded rebars coated by anti-corrosive materials in polymer cement mortar. Constr. Build. Mater..

[20] Ministry of Development, Public Works and Administration (2023). Code for the Production of Concrete and Execution of Works in Concrete, Reinforced Concrete and Prestressed Concrete—Part 1: Concrete Production (NE 012/1-2022).

[21] Romanian Standards Association (ASRO) (2021). SR EN 206+A2:2021—Concrete. Specification, Performance, Production and Conformity.

[22] Dong Z., Zheng S., Jiao L., Xu X., Yao Y., Gao Z., Li H. (2024). Experimental and parametric analysis of pull-out resistance of notched T-perfobond connectors. Appl. Sci..

[23] Zheng S., Zhao C., Liu Y. (2019). Analytical model for load–slip relationship of perfobond shear connector based on push-out test. Materials.

[24] Yankelevsky D.Z. (2024). The uniaxial compressive strength of concrete: Revisited. Mater. Struct..

[25] Padmanabham K., Rambabu K. (2022). Static pullout tests on retrofitted anchorage system in concrete using supplementary reinforcement. Saudi J. Civ. Eng..

[26] Zhou Z., Li Q., Liu Y. (2018). Dynamic response of concrete under high strain rates using explicit finite element method. Int. J. Impact Eng..

[27] Comité Européen de Normalisation (CEN) (2004). Eurocode 2: Design of Concrete Structures—Part 1-1: General Rules and Rules for Buildings (EN 1992-1-1:2004).

[28] Romanian Standard (1992). SR EN 1992-1-1, Eurocode 2: Design of Concrete Structures.

[29] Riedel W., Thoma K., Hiermaier S. Penetration of reinforced concrete by BETA-B-500 warheads. Proceedings of the 18th International Symposium on Ballistics (ISIEMS).

[30] Riedel W. (2004). Beton unter Dynamischen Lasten: Meso- und Makromechanische Modellierung.

[31] Riedel W., Mark K., Thoma K. (2009). Assessment of concrete structures under impact loading using numerical simulations. Int. J. Impact Eng..

